# Role of platelet parameters in *early detection and prediction of severity of* preeclampsia: A comparative cross-sectional study at Ayder comprehensive specialized and Mekelle general hospitals, Mekelle, Tigray, Ethiopia

**DOI:** 10.1371/journal.pone.0225536

**Published:** 2019-11-21

**Authors:** Feven Tesfay, Mikias Negash, Jemal Alemu, Mohammedtahir Yahya, Gebre Teklu, Meseret Yibrah, Tsegay Asfaw, Aster Tsegaye

**Affiliations:** 1 Department of Medical laboratory Sciences, College of Health Sciences, Mekelle University, Mekelle, Ethiopia; 2 Department of Medical Laboratory Sciences, College of Health Science, Addis Ababa University, Addis Ababa, Ethiopia; 3 Department of Gynecology and obstetrics, College of Health Science, Mekelle University, Mekelle, Ethiopia; Ospedale dei Bambini Vittore Buzzi, ITALY

## Abstract

**Background:**

Platelet parameters alterations are one of the most commonly identified hematological changes in preeclampsia (PE). However, their functions as a tool for prediction and prognosis of PE have not been extensively studied in developing countries. The aim of this study was to compare platelet count (PC), and platelet indices (mean platelet volume (MPV), platelet distribution width (PDW), and platelet large cell ratio (PLCR)) between preeclamptic and normotensive (NT) pregnant women and assess their role in diagnosis and prediction of PE development.

**Methods:**

A cross sectional comparative study was conducted at Ayder comprehensive specialized hospital (ACSH) and Mekelle general hospital (MGH) from January to March 2017. Platelet parameters of mild preeclamptic (mPE) (n = 35), severe preeclamptic (sPE) (n = 44) and NT pregnant women (n = 140) were analyzed using SYSMEX-XT 4000i automated hematology analyzer. One-way ANOVA supplemented with post-hoc test, receiver operating characteristics (ROC) curve and pearson correlation test statistical analyses were performed. *P* < 0.05 was considered significant.

**Result:**

Pregnant women with sPE had lower PC as compared with that of mPE and NT women (p<0.05). All platelet indices showed significant increment with severity of PE. PC was negatively correlated with platelet indices. There was a positive correlation among platelet indices. ROC analysis revealed that MPV had the largest area under the ROC curve (0.85; 95%CI (0.79, 0.89)) with cutoff value >9.45fl, sensitivity of 83.5%, specificity of 86.4%, positive predictive value of 77.6% and negative predictive value of 90.3%.

**Conclusion:**

MPV and PC were identified as good candidates for sPE diagnosis. Because evaluation of platelet parameters is rapid, reliable and economical, they can be utilized as an alternative biomarker for prediction and prognosis of PE.

## Background

The primary well-known function of platelets (PLTs) is hemostasis or prevention of bleeding during the earlier stages of clot formation [1, 2]. Platelet count (PC) and platelet indices, such as mean platelet volume (MPV), platelet distribution width (PDW), and large cell ratio (PLCR), are a group of derived platelet parameters obtained from automatic complete blood count [3]. Platelet parameters are suggested as a good candidate for diagnosis and prognosis preeclampsia (PE) [4].

PE is a multi-systemic pregnancy complication, characterized by the presence of high blood pressure and proteinuria after the 20^th^ week of pregnancy. It is considered as one of the major health problems associated with pregnancy and one of the causes of maternal mortality worldwide with prevalence of 5–8% [5–8]. In developing countries like Ethiopia, its prevalence ranges from 1.8 to 16.7% [9].

Even though the exact pathogenesis of PE remains unknown, placental vascular under-perfusion, maternal endothelial damage and increased vascular permeability are thought to contribute to the pathophysiology of the disease [10]. The injured endothelium due to defective placental trophoblastic invasion leads to activation of PLTs [11]. The activated PLTs contact the coagulation system leading to increase consumption as well as bone marrow production of platelets [12]. As a result, bone marrow releases young PLTs which are larger in size resulting in increased platelet indices such as MPV, PDW and PLCR [13].

The PC and MPV are determined during a routine automatic whole blood count and are commonly available parameters nowadays [14, 15]. This eases the indirect measure of the rate of platelet production and activation by calculating the indices from the result of the automatic whole blood count. Utilization of these simple markers could therefore facilitate early detection of maternal and fetal complications thereby could play a role as prognostic tool in the management of PE [16]. The aim of this study was to compare the platelet parameters in PE and NT pregnant women and evaluate the predictive significance of these parameters in determining the risk and severity of PE.

## Methods

### Study area and design

A cross-sectional comparative study was carried out from January to March 2017 at ACSH and MGH which are found in Mekelle City, the capital city of Tigray region. Mekelle is located in the northern part of Ethiopia, 783 km away from the capital city Addis Ababa.

### Sample size determination and sampling procedure

A formula of hypothesis testing for two population means was used to determine the initial sample size for the PE groups. Anticipated MPV and standard deviations for PE (10.15 and 1.10, respectively) and normal pregnancy (9.48 and 0.87, respectively) of a study done in Sudan [17] were used for this calculation. Using 0.2 of β, 5% of margin of error, 95% confidence level, 15% non-response rate and the aforementioned means and standard deviations, the initial sample size for PE (case) group was 40. A 1:2 allocation ratio was utilized to determine the number of NT pregnant women (control) group. This ratio is believed to evaluate the clinical accuracy of platelet parameters in PE. Accordingly, 120 were taken as initial sample size for the study. A total of 219 (79 cases of preeclamptic and 140 NT) pregnant women were then included using convenient sampling techniques during the data collection period.

### Data collection

Pregnant women presented to the respective hospitals for their own routine follow up checkups. They were clinically examined by experienced gynecologist (physicians) and categorized as preeclamptic or normal by making diagnosis based on brief clinical history, blood pressure (BP), and urine examination for protein. Cases were termed PE when there was high blood pressure (systolic blood pressure ≥140 mmHg, or diastolic blood pressure ≥90 mmHg, repeated two times four hours apart), proteinuria (≥0.3 g/dl), edema and other major symptoms (head ache, blurred vision, right upper quadrant pain) as early as the 20^th^ gestational week. PE cases were considered mild when the BP was ranged 140/90-160/110 mmHg without the major symptoms and severe when BP was ≥160/110 mmHg with or without the major symptoms and 140/90-160/110 mmHg with the major symptoms. Healthy pregnant women attending for antenatal care were taken as a control group. Pregnant women with intra-uterine fetal death, poor past obstetric history (such as recurrent miscarriage, pre-term labor, intrauterine growth restriction), gestational or insulin-dependent diabetes, heart, renal or hepatic dysfunction, inflammatory, and hematological diseases were excluded from the study.

Following standard operating procedures (SOPs), blood samples (3-mL) were collected from the study participants by qualified laboratory technologists using vacutainer tubes (purple cap) containing 2.0 mg/mL ethylenediamine tetraacetic acid (EDTA-K2). The collected blood samples were thoroughly mixed to avoid clump and clot formation. Obstetrics history (age and gestational age) of both case and control groups were also obtained from the medical records using predesigned data collection format.

### Laboratory analysis

The value of the platelet parameters of the samples were determined using hematology auto analyzer (SYSMEX-XT 4000i, Sysmex Corporation, Kobe, Japan) within 2 hours of blood collection. Hematology analyses were performed according to the hydro dynamic focusing (DC Detection), flow cytometer (using a semiconductor laser) and traditional impedance technology to improve accuracy of very low and very high PLT counts technology. The performance of the instrument was monitored by running three levels of controls and samples were analyzed only when the controls are in the accepted range for all parameters.

### Data analysis and interpretation

Data was entered and analyzed using Statistical Package for Social Science (SPSS) version 20 (SPSS INC, Chicago, IL, USA). The Mean and Standard deviation were used to summarize platelet parameters. One-way ANOVA supplemented with post-hoc test was used to compare the difference in mean platelet parameters across the three (NT, mPE and sPE) groups. ROC curve analysis was performed to calculate sensitivity, specificity, positive predictive value (PPV), and negative predictive value (NPV) for a given platelet parameter and indices (PC, MPV, PDW and P-LCR) in predicting PE. Pearson correlation test was used to investigate the relationship among continuous variables. A P-value of <0.05 was considered as statistically significant.

## Ethical considerations

The study was conducted after the study protocol was ethically reviewed and approved by the Department of Medical Laboratory Science Research and Ethical Review Committee (DRERC), College of Health Sciences, Addis Ababa University. After approval of ethical issues, permissions were obtained both from Tigray health bureau and the authorities of the hospitals to undertake the study. The aim, risk and benefit of the study as well as their right for withdrawal from the study at anytime were verbally explained for the study participants using local language in the study area and their informed verbal consent was obtained prior to data and sample collection. Samples were coded and confidentiality of patient data was maintained throughout the study.

## Results

This study included two groups of pregnant women: the control group included 140 NT pregnant women and case group was composed of 79 pregnant women with PE. Out of 79 preeclamptic cases, 35(44.3%) and 44(55.7%) were mild PE (mPE) and severe PE (sPE) respectively.

### Clinical characteristics of study participants

Characteristics of preeclamptic and NT pregnant women are presented in Table 1. While a significant difference was not found among the mean ages of mPE, sPE and control groups (*p>0*.*05*), gestational age of preeclamptic patients was found to be lower than the NT pregnant women (*p< 0*.*05*). The mean±SD BP of NT, mPE & sPE study participants was systolic 102.50±5.1 and diastolic 66.93± 8.03, systolic 143.14±5.29 and diastolic 97.71±6.17, diastolic 94.61±3.99 and systolic 157.95±16.07, respectively.

**Table 1:** Clinical characteristics of pregnant women with and without PE at ACSH and MGH, Mekelle, Ethiopia, from January–March 2017 (n = 219).

	Preeclampsia			P-value
	Normal (n = 140)	Mild (n = 35)	Severe (n = 44)	
**Age**	25.64±3.9	25.20±3.5	25.64±5.26	0.85
**Gestational age (in weeks)**	35.99±3.58	34.51±4.48	34.61±3.99	0.031

### Comparison of platelet parameters across severity of PE

Mean and standard deviation of platelet parameters of the three groups are shown in Table 2. Severe PE groups had lower PC and higher MPV, PDW and PLCR as opposed to mPE and Control groups (*p* ˂0.05). The average platelet parameters showed a significantly difference among the categories of women. Post-hoc tests also revealed significant variation of mean platelet parameters among each pairs of severity of PE.

**Table 2:** Comparison of platelet parameters by severity of PE among pregnant women attending ACSH and MGH, Mekelle, Ethiopia, January-March 2017 (n = 219).

Platelet parameters	Preeclampsia			P-value*
	Normal(n = 140)	Mild(n = 35)	Severe (n = 44)	
	Mean±SD	Mean±SD	Mean±SD	
PC(10^9^/L)	291.6±58.4	226±56.5	185.3±60.2	<0.001
MPV(fl)	8.4±0.9	11.5±2.1	12.3±1.7	<0.001
PDW(fl)	10.8±1.8	11.1±1.6	14.3±3.4	<0.001
PLCR(%)	29.1±6.8	30.8±6.6	35.3±8.9	<0.001

Mean (Standard Deviation); F-test (One way-Anova) for mean rank difference across severity of PE; PC-Platelet Count; MPV-Mean Platelet Volume; PDW-Platelet Distribution Width; PLCR-platelet large cell ratio; fl- femtolitre.

* (P-value is <0.001).

### Correlation between the platelet parameters by severity of PE

PC was negatively correlated with MPV, PDW and PLCR in NT pregnant women. For example, decrease in PC was proportional with increase in MPV (r = -0.35; p = 0.001). In pregnant women with mPE, none of the platelet parameters was significantly correlated. Whereas, PC was negatively correlated with PDW and PLCR in sPE (p<0.05) (Table 3).

**Table 3:** Correlation between platelet parameters in NT, mPE and sPE pregnant women visiting ACSH and MGH, Mekelle, Ethiopia, January–March 2017 (n = 219).

Platelet parameters	Preeclampsia		
	Normal (r)	Mild (r)	Severe (r)
**PC vs. MPV**	-0.35***	0.12	-0.16
**PC vs. PDW**	-0.36**	0.22	-0.32*
**PC vs. PLCR**	-0.38**	-0.1	-0.39**

r: Pearson correlation,

*** (P-value is <0.001),

**(P-value is <0.01),

*(P-value is <0.05)

PC-Platelet Count; MPV-Mean Platelet Volume; PDW-Platelet Distribution Width; PLCR-platelet large cell ratio

### Correlation between PC and platelet indices in women with PE

**Fig 1:**
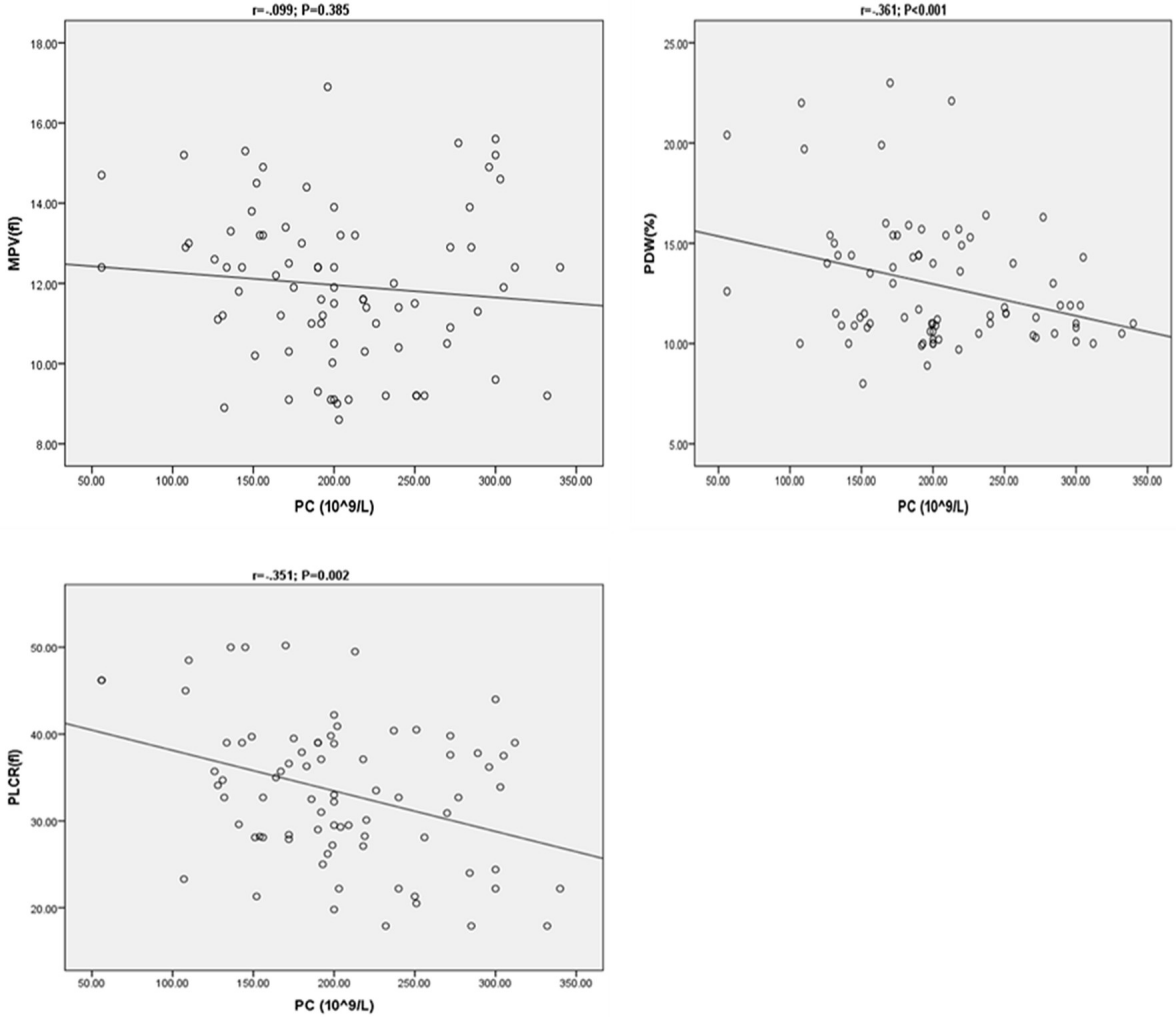
Correlation between PC and MPV, PDW, PLCR in women with PE (where; r = correlation coefficient). PC-Platelet Count; MPV-Mean Platelet Volume; PDW-Platelet Distribution Width; PLCR-platelet large cell ratio.

In preeclamptic participants, PC showed a significant negative correlation with PDW and PLCR and non- significant correlation with MPV (Fig 1).

### Comparison between the diagnostic values of platelet parameters

**Fig 2:**
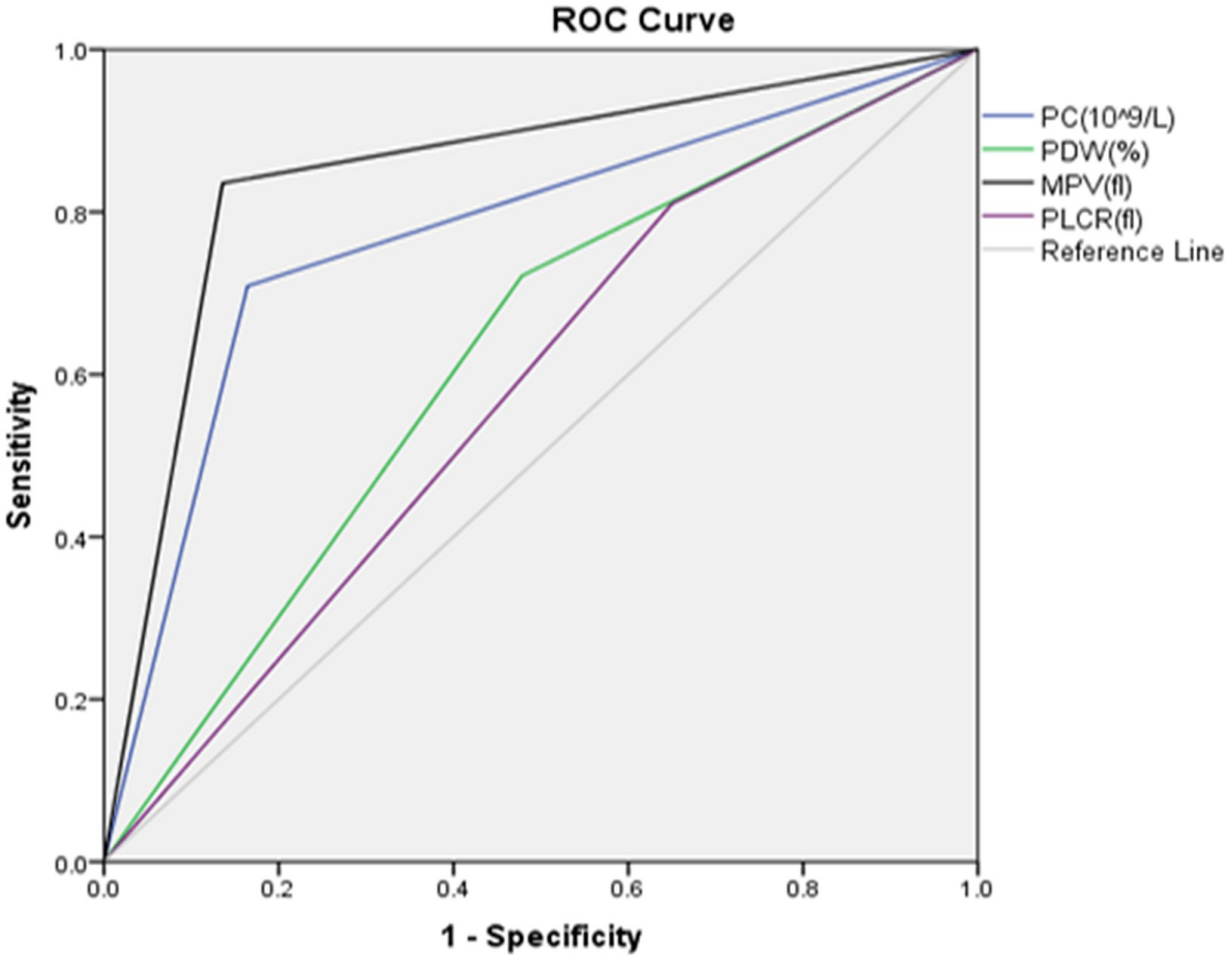
ROC curve of platelet parameters in pregnant women with PE visiting ACSH and MGH, Mekelle, Ethiopia, January-March 2017 (n = 219). PC-Platelet Count; MPV-Mean Platelet Volume; PDW-Platelet Distribution Width; PLCR-platelet large cell ratio.

In this study, ROC analyses were performed to identify the optimal cut-off levels of platelet parameters which were used to predict the development of PE (Fig 2 and Table 4). Accordingly, the analysis showed that PC can differentiate NT pregnant women from preeclamptic pregnant women at a cut off value <233x10^9^/L with sensitivity of 70.9% and specificity of 83.9%. MPV can differentiate NT pregnant women from preeclamptic pregnant women at a cut off value >9.45fl with sensitivity of 83.5% and specificity of 86.4%. PDW and PLCR had sensitivity and specificity of 72.2%, 52.1%, and 81.0%, and 35.0% at cut off values of >10.85fl and >26.2%, respectively. MPV had the largest area under the ROC curve (AUC = 0.85; 95%CI (0.79, 0.89)) followed by PC (AUC = 0.77; 95%CI (0.71, 0.83) (Fig 2 and Table 4).

**Table 4:** Comparison between the diagnostic values of platelet parameters of the study participants in ACSH and MGH, Mekelle, Ethiopia, January-March2017 (n = 219).

Platelet indices	Sensitivity (%)	Specificity (%)	PPV (%)	NPV (%)	Cut-off	AUC(95% CI)
**PC (10^9^/l)**	70.9	83.9	70.9	83.6	<233	0.77(0.71, 0.83)
**MPV (fl)**	83.5	86.4	77.6	90.3	>9.45	0.85(0.79, 0.89)
**PDW (fl)**	72.2	52.1	46.0	76.8	>10.85	0.625(0.557, 0.686)
**PLCR (%)**	81.0	35.0	41.3	76.6	>26.2	0.58(0.52, 0.639)

PC-Platelet Count; MPV-Mean Platelet Volume; PDW-Platelet Distribution Width; PLCR-platelet large cell ratio; fl- femtolitre.

## Discussion

PE is one of the significant causes of maternal and fetal mortality and morbidity in developing countries [5]. Widely available and cost-effective CBC parameters such as platelet count and platelet indices (MPV, PDW and PLCR) are suggested to be useful to predict the prognosis of PE [13].

In the present study, PC showed a significant decrease in sPE and mPE groups compared to control group. In other words, PC significantly declines in accordance with the severity of PE. This appears to be in consistent with the the pathophysiology of PE, which states that an endothelial activation induced increase in platelet aggregation and consumption resulting in decrease PC [18, 19, 20]. Similar finding of indirect relationship between PC and severity of PE were also reported by other studies [4, 13, 21, 22], which further supports that PC could be used as a candidate to predict PE.

ROC analysis demonstrated PC<233x10^9^/L as an optimal cutoff value with sensitivity of 70.97% and specificity of 89.3% to predict PE development. This result is relatively closer to the study by Eman *et al* (2013) [23], which reported a cut off value of < 198,000 (with 90% sensitivity and 92% specificity) to differentiate NT for mPE pregnant women and a cut off value of < 149,000 (with 84% sensitivity and 92%specificity) to differentiate mPE from sPE groups. However, our finding is inconsistent with the reports of other studies [17, 24, 25]. This variation could be explained by the difference in the method of measurement of these platelet indices.

Due to increased consumption and destruction of platelets, bone marrow produces and release large PLTs leading to increase MPV in PE [4]. However, literatures reported conflicting results regarding the relation between MPV and PE. For instance, Dadhich *et al* (2012), reported MPV (with cutoff of 8.5 fl, sensitivity 78%, and specificity 86%) as a good marker of platelet dysfunction in PE [21]. In our study there was also a significant increase in MPV from NT pregnant women [8.4±0.9fl] to mPE [11.5±2.1fl] and sPE patients [12.3±1.7]. Howarth S *et al* [26] reported that a combination of reduced PC and elevated MPV produced a sensitivity of 90% and a specificity of 83.3% when they were used to predict PE. Similar findings were also revealed by Hutt *et al* [27], indicating an increase in MPV two weeks prior to the occurrence of BP increment and development of other clinical features of PE. On the other hand, Cyehan *et al* [28]and Amita *et al* [20] did not find a significant difference in the MPV between PE and NT pregnant groups. ROC curve analyses of this study produced a MPV >9.45 cut off value with a sensitivity of 83.5% and specificity of 86.4%, which supports the results suggesting MPV being as a good marker for predicting PE.

The ability of PDW to differentiate preeclamptic from normal pregnant women at a cut off value ≥ 10.8 fl had an AUC of 0.625, sensitivity of 72% and specificity of 52%. Our finding agrees with other studies [13, 20, 21, 22, 29, 30] which demonstrated an increase in PDW in PE group as compared to normal pregnant group. The increased PDW could be explained by an increase in platelet turnover following decrease in platelets survival time as a result of increase in platelets destruction. Increase in bone marrow activity of unknown stimulus could also contribute to the observed high PDW [31]. Hence, an increase in PDW during PE could be considered as one of the important indicators to predict disease severity.

PLCR was significantly higher in the order of sPE, mPE and control groups. PLCR>26.2% (81% sensitivity, 35% specificity and 0.58 AUC) was observed to be an optimal cut off value to differentiate preeclamptic from normal pregnant women. This finding is consistent with the results of studied elsewhere [4].

Correlation analysis revealed that PC was negatively correlated with MPV, PDW and PLCR in NP women in this study. For instance, decrease in PC was proportional to an increase in MPV (r = -0.35; p = 0.001). None of the platelet parameters had significant correlations in women with mPE. Whereas, PC had a significant negative correlation with PDW and PLCR in women with sPE. The highest negative correlation index was found between PC and PLCR in sPE patients. A recent study by Sitotaw *et al* from the northern part of Ethiopia enrolling 33 mPE, 30sPE and 63 healthy pregnant women revealed an increase in MPV, PDW, P-LCR with the advancement of PE. Whereas, PC decreased with the severity of the disease [32]. Platelet parameters are among the least biomarkers being utilized by clinicians in Ethiopia despite the wide availability of automated hematology analyzers in the country [33]. One study [34] reported a statistically significant negative correlation between PC and the platelet indices in case of hyper-destruction thrombocytopenia, which is the main cause of decrease in PC in PE, underscoring the utility of such simple markers in the clinical management of such patients.

Our study has certain limitations. Being cross-sectional study, establishing the role of platelet parameters in prediction and assessing the severity of PE during the whole pregnancy period was not possible. Further longitudinal studies which could evaluate the platelet count and other platelet parameters at various gestational ages of pregnancy are required to confirm the role of these parameters in diagnosing and predicting severity of preeclampsia.

## Conclusions

This study demonstrated significant decrease in PC and increase in platelet indices during PE. This shows that PC, MPV, PDW and PLCR could be used as supplementary, easy, reliable, economic and rapid biomarkers for detection of PE and assessment of its severity.
